# Ethnobotanical and Ethnopharmacological Study of *Paris polyphylla* var. *yunnanensis* in Yunnan Province, China

**DOI:** 10.3390/plants13202914

**Published:** 2024-10-18

**Authors:** Xiuxiang Yan, Angkhana Inta, Ge Li, Hataichanok Pandith, Terd Disayathanoowat, Lixin Yang

**Affiliations:** 1 Key Laboratory of Economic Plants and Biotechnology, Kunming Institute of Botany, Chinese Academy of Sciences, Kunming 650201, China; yanxiuxiang@mail.kib.ac.cn (X.Y.); lllyy18@126.com (G.L.); 2 Department of Biology, Faculty of Science, Chiang Mai University, 239 Huay Kaew Road, Chiang Mai 50200, Thailand; aungkanainta@hotmail.com (A.I.); hataichanok064@gmail.com (H.P.); 3 School of Ethnic Medicine, Yunnan Minzu University, Kunming 650504, China; 4 Research Center of Deep Technology in Beekeeping and Bee Products for Sustainable Development Goals (SMART BEE SDGs), Chiang Mai University, 239 Huay Kaew Road, Chiang Mai 50200, Thailand

**Keywords:** *Paris polyphylla* var. *yunnanensis*, traditional Chinese medicine, traditional medicinal knowledge, ethnopharmacology, folk doctor

## Abstract

The traditional medicinal knowledge in the northwest of Yunnan Province, China have been poorly studied. *Paris polyphylla* var. *yunnanensis* (PPvY) is widely cultivated and used as indigenous traditional Chinese medicine (TCM) to treat cancer in northwest Yunnan. This study aims to reveal the traditional medicinal knowledge of PPvY and folk formulas related to PPvY through literature research and ethnobotanical investigation. Semi-structured interviews were conducted with 14 highly regarded folk doctors in the northwest of Yunnan, China, based on relevant data collected in the initial phase of the research. We identified twenty-three traditional treatments, thirty pairing herbs used with PPvY in therapy, and eight processing methods of PPvY. The results indicated that PPvY and its associated formulas containing PPvY were primarily used for treating cancer and inflammation and for clearing heat and detoxifying. The TCM herbs most frequently used alongside PPvY included *Engleromyces sinensis* and *Glycyrrhiza yunnanensis*. The commonly employed processing methods primarily involved using PPvY in both its dry and fresh forms, while special processing methods, such as processing in wine and honey, steaming, and foil-packet boiling, were worth further research. Our results highlight the diversity of medicinal plants and the richness of traditional medical knowledge in northwest Yunnan, China. This study may offer clues for the development and research of indigenous medicinal plants. Additionally, a collective effort is needed to create a plan for the sustainable use of indigenous medicinal plants, enhancing local economic development while safeguarding biodiversity and traditional medicinal knowledge.

## 1. Introduction

The genus *Paris*, also known as “*Chonglou*” in Chinese, includes perennial herbaceous plants which is mainly distributed in various regions of Yunnan, as well as in Provinces such as Sichuan and Guizhou [1,2]. The *Paris* plant is a member of the Melanthiaceae family encompassing 26 species based on current taxonomy [1]. The dried rhizome of *Paris polyphylla* var. *yunnanensis* (Franch.) Hand.–Mazz. (PPvY) and *Paris polyphylla* Smith var. *chinensis* (Franch.) Hara are recorded in Chinese Pharmacopoeia (2020 version) [2] and Yunnan Province Drug Standards [3], which have the effects of clearing heat and detoxifying, reducing swelling and pain, and cooling the liver and calming convulsions. There are many species of the genus *Paris* distributed in Yunnan, among which PPvY stands out as one of the important varieties of *Paris polyphylla* Smith in traditional Chinese medicine (TCM) produced in Yunnan. With breakthroughs in seedling-breeding technology, a surge in PPvY planting has occurred across the country in recent years, which was greatly alleviated the supply–demand contradiction of PPvY resources. In 2015, the Good Agriculture Practice (GAP) base for PPvY became the only certified *Paris*-genus-planting base in China. In 2016, “Yulong PPvY” was awarded the Geographical Indication Registration Certificate for Agricultural Products by the Ministry of Agriculture of China, making it the first *Paris* product to receive this prestigious recognition. In 2018, “Yulong PPvY” in Yulong County, Lijiang City, Yunnan Province was recognized as one of the first batch of 43 characteristic agricultural product advantage areas in Yunnan. Yunnan is indeed the original and primary production area of PPvY, with the planting area and yield accounting for over 90% of the country. In 2020, the land area dedicated to PPvY in Yunnan reached 180,000 acres. Overall, PPvY was primarily cultivated in the northwest region of Yunnan Province [3,4,5].

The northwest of Yunnan is situated in the core area of the Hengduan Mountain range and is recognized as one of the areas with the richest biodiversity in China [6]. The northwest region of Yunnan serves not only as a reservoir of medicinal plant germplasm resources and a refuge for rare and endangered medicinal plants but also as a site for the introduction and domestication of wild medicinal plants. Additionally, it represents a cultural intersection among Yunnan, Sichuan, and Tibet [7,8,9]. Nine ethnic groups—Bai, Naxi, Miao, Lisu, Yi, Tibetan, Hui, Lahu, and Pumi—in this region have long-term interactions with the plants in their surrounding environment, leading to distinct cultural practices regarding plant utilization among these groups [10]. The concept of co-evolution between biodiversity and cultural diversity has gained broad acceptance within the international community [11]. Ethnic medicine culture is one of the five aspects of ethnic culture. Traditional medical knowledge acts as a vehicle for expressing this cultural heritage and represents a treasure trove of knowledge accumulated by humanity for disease prevention and treatment [10]. Notably, TCM has made significant contributions in combating Severe Acute Respiratory Syndrome (SARS) in 2003 and Corona Virus Disease 2019 (COVID-19) in 2020, indicating that traditional medical culture plays a crucial role in combating diseases and should not be underestimated [12,13,14,15,16,17].

The diversity of regional plants and ethnic groups makes their traditional knowledge extremely diverse [18,19,20]. Most ethnobotany research focuses on investigating and cataloging medicinal plants within specific regions [16,21,22,23,24,25,26]. However, a framework has been established to integrate cross-cultural ethnobotanical knowledge at the genus level, encompassing species diversity, cultural diversity, and traditional uses [27,28]. However, traditional medical knowledge mainly focuses on personalized treatment administered by folk herbalists tailored to the current condition of the disease. Typically, this treatment involves a unique combination of TCM practices, reflecting the various TCMs required for the patient’s physical recovery [15]. Folk doctors frequently modify their treatment based on the patient’s symptoms, disease status, and constitution. As a result, TCM formulas exhibit unique individuality and may include multiple herbs together, sometimes comprising up to twenty different types [9,29,30,31]. The combination of herbs can enhance the effectiveness of individual herbs, treat additional symptoms, or reduce side effects [9,29,31]. The synergistic effects achieved through the use of multiple herbs are often regarded as superior to those of a single herb [15]. However, in northwest Yunnan, an area rich in traditional medical knowledge and culture, treatment formulas associated with PPvY have yet to be collected and organized. Additionally, the processing methods, compatibility of medicinal herbs, and unique efficacy of these formulas have not been systematically analyzed.

This study investigates the clinical application of PPvY by folk doctors in the northwest of Yunnan, as well as the relevant literature and books, including processing methods, compatibility of medicinal herbs, and traditional efficacy. By exploring the traditional knowledge of folk herbal medicine, understanding the traditional ethnic medical knowledge related to the traditional ethnic culture of PPvY may help reveal new methods and research directions for further scientific inquiry.

## 2. Results and Discussion

### 2.1. Traditional Efficacy of PPvY

There were 23 traditional treatments of PPvY mentioned by folk doctors, which were listed in Table 1. According to the indicating diseases and classification of diseases, traditional treatment with formulas containing PPvY could be divided into cancer, anti-inflammatory, clearing heat and detoxifying, respiratory system diseases, skin and subcutaneous tissue diseases, and other types of diseases. The most common disease for PPvY use was inflammation, with twelve out of fourteen folk doctors indicating that they had included PPvY in anti-inflammatory treatment. The folk doctors (D4, D6, D11, and D12) used PPvY to treat all types of inflammatory in association with TCM *Engleromyces sinensis* M.A. Whalley, Khalil, T.Z. Wei, Y.J. Yao and Whalley. Doctor D5 used PPvY in combination with TCM herbs *E. sinensis* and *Ephedra likiangensis* Florin to treat all types of inflammation, while doctor D9 treated periodontitis with the same herbal combinations. Four out of fourteen folk doctors (D8, D9, D10, and D11) indicated that they had included PPvY in treatment of lung cancer. There were three folk doctors (D8, D9, and D10) who used the formulas containing PPvY to treat liver cancer. However, only one folk doctor (D11) reported using a formula containing PPvY for gastric cancer. In addition to the three types of cancer mentioned above, folk doctors D8 and D10 showed that other types of cancer could also be treated using formulas containing PPvY. The powder from PPvY (20 g), *Scleromitrion diffusum* (Willd.) R. J. Wang (50 g), *Scutellaria barbata* D. Don (50 g), and *E. sinensis* (20 g) was prepared as a decoction to treat all types of cancer, especially for gastric cancer, which was mentioned by doctor D11. Both doctors D8 and D9 provided an heirloom prescription that can be used to treat liver and lung cancer. It is interesting that doctor D8 is the father of D9. There were 11 herbs in this traditional prescription, including PPvY (6 g), *Panax bipinnatifidus* Seem. (3 g), *Panax notoginseng* (Burkill) F. H. Chen ex C. H. Chow (1 g), *Fritillaria cirrhosa* D. Don (2 g), *Pleione bulbocodioides* (Franch.) Rolfe (1 g), *Psammosilene tunicoides* W. C. Wu and C. Y. Wu (0.2 g), *Panax quinquefolius* L. (1 g), *E. sinensis* (0.2 g), *Cynanchum otophyllum* C. K. Schneid. (1 g), *Glycyrrhiza yunnanensis* S. H. Cheng and L. K. Dai ex P. C. Li (1 g), and *Gastrodia elata* Bl. (2 g). Doctor D8 employed PPvY to treat both lung and liver cancer associated with *E. sinensis* and *P*. *bulbocodioides*. Doctor D8 used PPvY combined with *E. sinensis* and *Anemone rivularis* Buch.–Ham. ex DC. to treat metro carcinoma. Doctor D10 mentioned that PPvY mixed with *E. sinensis* in a 1:1 ratio (m/m) is used to treat all types of cancer. The TCM combination of PPvY and *Ostrea gigas* Thunberg was used to treat lung cancer by doctor D11. Doctor D14 mentioned a prescription related to PPvY for treating tumors and nodules, including breast nodules and uterine fibroids. This prescription comprised eight TCM herbs, including *F*. *cirrhosa*, *Pa. bipinnatifidus*, PPvY, *E. sinensis*, *Angelica dahurica* (Fisch. ex Hoffm.) Benth. and Hook. f. ex Franch. and Sav., *Taraxacum mongolicum* Hand.–Mazz., *Ranunculus ternatus* Thunb., and *Prunella vulgaris* L. It is important to note that the proportions of each herb in some formulas were not provided due to confidentiality concerns. The four folk doctors (D8, D9, D10, and D11) mentioned above all indicated that cancer is related to inflammation. They all expressed the need for anti-inflammatory treatment while treating cancer. Therefore, PPvY is needed to clear heat and detoxify. Five folk doctors (D1, D5, D8, D9, and D10) suggested that PPvY could be used for clearing heat and detoxifying.

In respiratory system diseases, fourteen folk doctors mentioned a total of six traditional treatments related to PPvY, including pulmonary diseases, tonsillitis, sore throat, tracheitis, pharyngitis, and cold. Folk doctors D1 and D3 indicated that they used PPvY to treat pulmonary diseases. Both D2 and D11 used the formulas contained PPvY in the treatment of tonsillitis. Only one folk doctor reported that they had used a formula containing PPvY to treat these four diseases, including sore throat (D5), tracheitis (D7), pharyngitis (D7), and cold (D1). For skin and subcutaneous tissue diseases, there were five folk doctors (D3, D7, D8, D9, and D10) who usually treated traumatic injury using a formula containing PPvY. Four folk doctors (D6, D8, D9, and D10) reported that they had used the formula containing PPvY to treat furuncles, carbuncles, and abscesses. Only one folk doctor reported that they had used formulas containing PPvY to treat these three diseases: hemorrhoids (D7), ulceration (D6), and snake venom (D6). For other types of diseases, five folk doctors (D3, D4, D5, D6, and D11) indicated that they usually treated mumps using formulas containing PPvY. Only one folk doctor reported that they had used formulas containing PPvY to treat these five diseases: infantile syncope due to fright (D1), gastrointestinal disease (D1), thyroiditis (D3), gynecological diseases (D3), and clearing damp (D9). Importantly, the number of folk doctors who used traditional treatments with formulas containing PPvY is in the following order: anti-inflammatory (12); clearing heat and detoxifying (5); mumps (5); traumatic injury (5); furuncles, carbuncles, abscesses (4); lung cancer (4); liver cancer (3); all cancers (3); pulmonary diseases (2); tonsillitis (2).. The number of traditional diseases treated with formulas containing PPvY mentioned by each folk doctor was in the following order: D8 (7) = D9 (7) = D10 (7) > D3 (6) > D1 (5) = D6 (5) = D11 (5) > D5 (4) = D7 (4) > D2 (2) = D4 (2) = D14 (2) > D12 (1) = D13 (1). In summary, according to the method of administration, traditional treatments could be divided into two categories: topical and oral. The main external uses are for traumatic injury, mumps and furuncles, carbuncles, and abscesses. Oral administration mainly focused on cancer and respiratory diseases. Both external and internal use have a significant correlation with anti-inflammatory and heat-clearing detoxification.

These survey results on traditional efficacy are consistent with modern pharmacological and chemical ingredients studies of PPvY. Modern pharmacological studies have demonstrated that PPvY exhibits a wide range of biological activities, including anti-tumor effects, hemostatic properties, anti-inflammatory, and analgesic effects, as well as anti-fungal activity and other pharmacological properties [32,33,34]. However, steroid saponins are the primary chemical components responsible for anti-cancer activity [33,35]. There are 177 compounds in PPvY, including 112 steroidal saponins [35,36]. Our previous research verified that the total contents of Paris saponins I, II, and VII were 6.96% in the dried rhizomes of cultivated PPvY, reaching levels 10 times higher than Chinese Pharmacopoeia standards (0.6%). Six Paris saponins—Paris saponins I, II, VII, III, H, and Pb—were isolated and identified in cultivated PPvY with cytotoxicity against three human liver cancer cell lines (SMMC-7721, SK-HEP-1, and HepG2) and one human lung cancer cell line, A549. Six saponins induced significant cell apoptosis and cell cycle arrest in human cancer cells, which were associated with the loss of mitochondrial membrane potential [34]. In summary, these results for modern phytochemical and pharmacological experiments for PPvY are further validated and supported by traditional medicinal knowledge.

### 2.2. Paring Herbs Used with PPvY in Therapy

TCM generally emphasizes a complex system of multiple components, multiple targets, and their synergistic effects among components [15]. Based on treatment experience, folk doctors indicated that only formulas can be used rather than individual herbs when treating complex diseases such as inflammation and cancer. The results in survey showed that there were 30 TCM herbs used in formulas with PPvY (Table 2). Out of fourteen folk doctors, eleven mentioned that they had prescribed PPvY and *E. sinensis* together during treatment. Both these two herbs had similar properties for clearing heat and detoxifying. Additionally, *E. sinensis* had been traditionally recognized for its efficacy in anti-cancer, antioxidant, and anti-inflammatory properties, as documented in indigenous books and pharmacological studies [29,37,38,39]. The second most commonly paired herb was *G. yunnanensis*, which had been mentioned by six out of fourteen folk doctors. It is prescribed together with PPvY for balancing purposes. To the best of our knowledge, there were currently no pharmacological articles that reported the use of *G. yunnanensis* to treat cancer and other diseases. However, phytochemical research on *G. yunnanensis* was conducted as early as the 1990s [40,41,42]. The third most commonly paired herbs were *P. bulbocodioides*, *G. elata*, *Pa. bipinnatifidus*, which were all mentioned by four folk doctors and been prescribed together with PPvY. Modern phytochemical and pharmacological studies indicated that the extracts and new isolated compounds from *P. bulbocodioides* exhibit anti-cancer, hepatoprotective, anti-inflammatory, neuroprotective, and antioxidant properties [43,44,45,46]. There are currently phytochemical and pharmacological articles that report the use of *G. elata* to treat diseases related to sedative and hypnotic activities, neuroprotective effects, and conditions of the circulatory and cardio–cerebrovascular systems [2,47,48,49,50]. Modern phytochemical and pharmacological research report that the root and rhizome of *Pa. bipinnatifidus* could be used for their anti-tumor and anti-inflammatory properties, analgesic and sedative effects, as well as for the protection of the cardiovascular and cerebrovascular systems and liver health [2,51,52,53]. Two out of fourteen folk doctors mentioned that there were four TCM herbs that are commonly used in formulas together with PPvY, including *E. likiangensis*, *P. tunicoides*, *T. mongolicum*, and *A. rivularis*. To our knowledge, there were currently no pharmacological articles that reported and verified the anti-inflammatory and anti-cancer effects of *E. likiangensis* and *P. tunicoides*. Both PPvY and *T. mongolicum* were known for the effects of clearing heat and detoxifying while *T. mongolicum* was a famous medicinal and edible plant. Phytochemistry for *T. mongolicum* revealed that it had various bioactive ingredients, mainly including flavonoids, sterols, polysaccharides, phenolic acids, and volatile oils. Pharmacological research had demonstrated that polysaccharides from *T. mongolicum* possess a range of beneficial effects, including immunomodulatory, anti-inflammatory, antioxidant, anti-tumor, hepatoprotective, hypolipidemic and hypoglycemic, antibacterial, regulation of intestinal microbial, and antifatigue activities [54,55]. The cytotoxicity of saponins isolated and identified from *A. rivularis* has been evaluated against five human cancer cell lines, including HL-60, HepG2, A549, Hela and HSC-T6. Additionally, a new triterpene ester compound isolated from *A. rivularis* has been reported to exhibit antimicrobial activity [56,57,58]. The remaining 21 TCM herbs were only mentioned by one folk doctor to be used in formulas with PPvY. The number of paired TCM herbs in formulas with PPvY mentioned by each folk doctor was in the following order: D9 (10) = D10 (10) > D14 (7) > D6 (5) = D7 (5) = D8 (5) > D5 (4) > D3 (3) > D1 (2) = D4 (2) = D11 (2) > D2 (1) = D12 (1) = D13 (1). In summary, the folk doctors for D9 and D10 reported the most traditional treatments and paired herbs with PPvY. The TCM herbs with higher frequency of use were *E. sinensis* (11), *G. yunnanensis* (6), *P. bulbocodioides* (4), *G. elata* (4), *Pa. bipinnatifidus* (4)*, E. likiangensis* (2), *P. tunicoides* (2), *T. mongolicum* (2), and *A. rivularis* (2). The TCM herbs *P. bulbocodioides*, *T. mongolicum*, *E. sinensis*, *E. likiangensis*, and *P. tunicoides*, when paired with PPvY, are used to clear heat and detoxify, according Chinese pharmacopeia and other indigenous ethnic medicinal books [2,9,29]. In summary, the combination with herbs is consistent with the traditional ethnic medicine culture, following the principles of enhancing efficacy, reducing toxicity, and balancing efficacy, which is consistent with modern TCM theory.

### 2.3. Preparation Method and Dosage of PPvY in Therapy

Some people usually believed that the active ingredients in fresh plants remain intact, making them the most effective [3,23,59]. Ten out of fourteen folk doctors often use fresh medicinal plants and rarely use dry plants. The surveyed folk doctors usually mixed several herbs together to form a prescription, rather than a single herb. In addition, folk doctors usually practiced a personalized form of medication by preparing doses based on individual patients during the treatment, rather than measuring consistent doses. The processing methods mentioned by folk doctors were in the following order: use in the fresh (10) > use in the dry (7) > processed with wine (6) > dry in the shade (5) > dry in the sun (3) = processed with honey (3) > steaming (2) > foil packet boiled (1) (Table 3).

Folk doctors usually used various additives, such as alcohol and honey, to enhance the efficacy of PPvY. The combination of TCM and alcohol has a long-standing history. The TCM is soaked in alcohol for approximately a month, after which the patient can experience therapeutic effects by either consuming the liquid or applying it to the affected area. Alcohol can serve as a solvent in place of water, allowing fresh or dried TCMs to be infused for consumption or external application. Doctors generally believed that alcohol extracts retain more active ingredients from medicinal plants than water, making them more effective in treating diseases. PPvY processed with honey was commonly used to treat respiratory system disease, as honey was widely believed to relieve cough and moisten the lungs. To the best of our knowledge, there are currently no scientific articles that report the potential mechanism for treating diseases by the combination of PPvY and honey. A hypothesis has been proposed that the microbes presented in honey may interact with the active compounds in PPvY, potentially reducing toxicity and enhancing its efficacy. However, scientific experiments are needed to further validate this hypothesis. The reason for and efficacy of processing in both wine and honey had a significant correlation with their anti-inflammatory properties and their ability to clear heat and detoxify. The results of our previous study found that an alcohol concentration of 50% to 80% was effective for extracting much more active saponins in PPvY, which had cytotoxic activity against human cancer cells [33,34,35]. The alcohol generally used by folk doctors was a traditional Chinese liquor, typically made from fermented grains, with an alcohol content of around 50%. These experimental results further verified the folk traditional medical knowledge. However, PPvY had two special processing methods, including steaming and foil-packet boiling. It is essential that PPvY was acquired in fresh form to process. According to doctors’ recollections, PPvY processed using these two methods had better therapeutic effects than other processing methods, particularly in terms of anti-cancer, anti-inflammatory, and heat-clearing and detoxifying effects. Perhaps during the steaming and boiling process, hydrophilic compounds such as polysaccharide were extracted, while other effective ingredients in PPvY were accumulated, which need further research.

In addition, both fresh and dried PPvY could be used as TCM. Drying methods for PPvY could also be carried out under sun or shade, which was closely related to the traditional knowledge of the folk doctors themselves. The number of processing methods with PPvY mentioned by each folk doctor was in the following order: D10 (6) > D3 (4) = D8 (4) > D2 (3) = D4 (3) = D6 (3) = D7 (3) = D9 (3) > D1 (2) = D5 (2) > D11 (1) = D12 (1) = D13 (1) = D14 (1). In summary, PPvY can be used in both its dried and fresh forms as TCM herbs. According to the method of administration, traditional treatments could be categorized into two types: topical and oral. For external use, there were two primary methods, including powder application and wiping with an alcohol preparation. In terms of oral administration, the two main methods were water decoction and taking in powder form. Only the dried form of PPvY could be taken in powder form, while both the fresh and dried forms could be used for water decoction.

### 2.4. Evaluation of Indigenous Knowledge on TCM through ICF Value

Fourteen informants reported treatments of ten different diseases, including lung cancer, uterine cancer, all types of cancer, various types of inflammation, pulmonary diseases, various nodules, infantile syncope due to fright, mumps, furuncles, carbuncles, abscesses, and traumatic injuries (Table 4). In Table 4, “all cancer” indicates that the specific type of cancer being treated was unclear to folk doctors, suggesting that TCM may be used to treat all types of cancer. The term “various nodules” encompassed pulmonary nodules, breast nodules, thyroid nodules, and others. Pulmonary diseases mainly included conditions such as cough, cold, pharyngitis, pulmonary fever, tuberculosis, cough relief, pneumonia, and others. The results of the Informant Consensus Factor (ICF) values indicated that the highest value among the four diseases—lung cancer, various nodules, furuncles, carbuncles, abscesses, and traumatic injuries—was 1. The ICF values were as follows: uterine cancer at 0.88, pulmonary diseases at 0.83, all cancers at 0.75, and various types of inflammation at 0.5. Both infantile syncope due to fright and mumps recorded the lowest ICF values of 0.00.

These varying ICF values suggested that these folk doctors share commonalities in their approaches to treating diseases, while also exhibiting their own unique medicinal practices. The larger the ICF value, the greater the difference among folk doctors, who have their own unique medicinal practices. However, PPvY can be used to treat these two types of diseases—infantile syncope due to fright and mumps—a view widely shared among informants. In other words, there was minimal variation in the treatment of these two diseases among the 14 folk doctors. Additionally, the ICF values for the other eight diseases were higher than 0.5, indicating that the types of TCM used for treating diseases (such as cancer, pulmonary, etc.) varied significantly among the folk doctors. Overall, these differences indicated the unique medicinal practices and traditional ethnic medicinal knowledge of folk doctors.

### 2.5. The Future Work to Medicinal Herbs and Traditional Knowledge

Our investigation results revealed that traditional medical knowledge was significantly threatened due to a lack of written records, conservative inheritance patterns, and a low level of interest among young people in traditional healing practices. In the future, traditional ethnic medicine should focus on the following three aspects of work. Firstly, a more comprehensive investigation should be conducted to record and catalog medicinal knowledge in the northwest of Yunnan using ethnobotanical methods. Secondly, scientific approaches such as pharmacology, metabolomics, network pharmacology, and molecular biology should be employed to elucidate the principles of ethnic medicine. The substance basis and potential pharmacological mechanisms of traditional ethnic TCM herbs were fully revealed through scientific methods, which was beneficial for the protection and sustainable development of traditional knowledge. Thirdly, it was necessary to protect the medicinal plant resources. There were two key aspects: first, promoting the cultivation of medicinal plants through scientific and technological support to reduce reliance on wild harvesting; second, suggesting implementing policies to promote access and benefit-sharing system of indigenous traditional medical knowledge. Specifically, establishing a fair and benefit-sharing relationship among holders, providers, and users of traditional knowledge not only safeguards the interests of local communities but also contributes to the protection of traditional medical knowledge.

## 3. Materials and Method

### 3.1. Study Area

The research sites of this study were located in the northwest of Yunnan Province, within the core area of the Hengduan Mountains—one of the most biodiverse regions in China. This area is not only a treasure trove of medicinal plant germplasm resources but also rich in ethnic culture, which has fostered unique traditional ethnic medicine knowledge [6,10]. The research area encompasses diverse ecosystems, including alpine, subalpine, wetland, and valley vegetation ecosystems. In addition, the vegetation ranges from subtropical to typical subtropical, temperate, alpine cold temperate, and cold tundra. These rich ecosystems and climate conditions have nurtured an abundance of medicinal plant resources [7,8].

**Figure 1:**
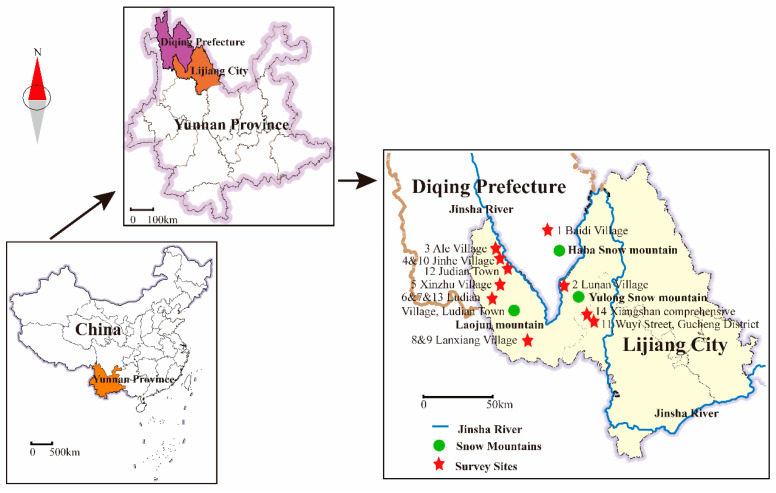
Location map of study sites in northwest Yunnan, China. The red star indicates the survey sites. The green circle indicates the location of the snow mountains. The blue lines represent the Jinsha River.

This study was conducted in Baidi village in Shangri La city and Lunan village, Ale village, Jinhe village, Xinzhu village, Ludian village, and Lanxiang village in Yulong County, all of which are located near the Jinsha River. These sites are situated near three major snow mountains—Haba Snow Mountain, Laojun Mountain, and Yulong Snow Mountain—and were selected for their representative characteristics of traditional medicine, including aspects of traditional economy, ethnic culture, ecosystems, climate, and geographical location (Figure 1).

### 3.2. Literature Review

The literature review was carried out on how many ethnic minorities use PPvY for treating diseases in China, particularly for cancer treatment. The effective folk formulas were collected and analyzed, drawing on existing clinical medication experience. Studies were searched from China National Knowledge Infrastructure (CNKI, https://pubmed.ncbi.nlm.nih.gov/ (accessed on 5 May 2021)), Web of Science (https://webofscience.clarivate.cn/wos/alldb/basic-search (accessed on 26 May 2021)), and National Center for Biotechnology Information (NCBI, https://pubmed.ncbi.nlm.nih.gov/ (accessed on 6 June 2021)). The literature research was completed in 2021 [32]. The results indicated that PPvY is widely used as medicinal herb by eight out of 56 ethnic minorities in China; the order ranked by the quantity of folk formulas include Yi, Naxi, Bai, Dai, Hani, Yao, Buyi, and Zang people. A total of 62 experientially effective formulas were found, including 29 simple formulas and 33 compound formulas. The main diseases treated, traditional processing methods, compatible herbs, and their respective doses have been discussed. Among these, Yi, Bai, Dai, and Naxi people used PPvY to treat cancer. Additionally, the collection and organization of folk formulas related to PPvY are ongoing, aiming to provide reference materials for future visits and field investigations.

### 3.3. Ethnobotanical Surveys

The focus of this ethnobotanical investigation was to understand local knowledge and practices related to PPvY in the northwest Yunnan region of China. Semi-structured interviews were conducted with 14 folk doctors with a big reputation in the northwest region of Yunnan, China, based on relevant data collected during the initial phase of the study. Traditional knowledge related to PPvY, including folk processing methods, traditional efficacy, and dosage, were collected. Furthermore, the main types of diseases treated and the TCM varieties used by folk doctors were also collected and analyzed.

Ethnobotanical investigation was conducted in villages surrounding the three snow mountains—Haba Snow Mountain, Laojun Mountain, and Yulong Snow Mountain—in northwest Yunnan, China from September to October 2023. The interviews were conducted in Chinese. According to the principles of ethnobotany, this study designed the following questions for interviews: (1) What are the main types of diseases that you excel at treating during the diagnosis and treatment process? Which TCM herb do you use for treatment? (2) What diseases do you treat with PPvY? Which ones are the most common? (3) What TCM herbs are commonly used in conjunction with PPvY? (4) What is the commonly used dosage when using PPvY? (5) What are the common side effects during the use of PPvY? How to avoid it? What kind of population do you not use PPvY in? (Children? Old man? A pregnant woman? Seriously ill patients?) (6) Is PPvY commonly used as a single herb or formula? Please provide a list of one or more formulations that you commonly used containing PPvY in a confidential manner.

The survey information form was provided to folk doctors before each interview, which included the purpose and methods of the survey, as well as how to use this information in the future and how to protect privacy. After the folk doctors reviewed the survey information form, permission was obtained through oral consent. Semi-structured interviews took place immediately following the consent of folk doctors, with each interview lasting approximately 30–60 min. The above six question served as guidance for the interview content, while allowing each folk doctor to expand based on responses. At the same time, follow-up questions may be raised at appropriate times, but some questions may be considered unnecessary. Folk doctors were also asked about the development and inheritance of ethnic medicine in the future. Each interview was recorded for review and translation into English. All of our ethnobotanical surveys were carried out with informed consent.

### 3.4. Quantitative Analysis

The Informant Consensus Factor (ICF) is a key indicator used for quantitative analysis in ethnobotany. The ICF value helps assess the degree of difference in the types of TCM used by different folk doctors when treating a certain type of disease. In this survey, each listed TCM was utilized to treat multiple types of diseases. Therefore, the ICF index [60] was utilized to assess the consistency of the information gathered from informants regarding the use of TCM. And the formula for calculating the ICF value was as follows:

$$ICF=\frac{\left(Nur-Nt\right)}{\left(Nur-1\right)}$$

where *Nur* is the sum of the number of TCM species used by all folk doctors to treat a specific type of disease, and *Nt* is the total number of the same TCM species mentioned by all folk doctors to treat the specific disease. The ICF value ranges from 0 to 1. The larger the ICF value, the greater the difference in the types of TCM used to treat a specific disease among folk doctors. The smaller the ICF value, the more concentrated the TCM species used by folk doctors to treat certain diseases.

### 3.5. Demographics of Folk Doctors

There are tens of thousands of TCM practitioners in China [15]. Folk doctors with a cultural background in ethnic medicine and over two generations of inherited medical knowledge were selected as our interviewees. Personal information, such as the name and workplace of folk doctors, was not recorded in this study in order to protect their privacy. Furthermore, folk doctors are required to discuss patient cases anonymously to ensure privacy. The folk doctors interviewed in this study were assigned identification numbers ranging from D1 to D14 (Table 5). Basic background information on the 14 folk doctors was collected, including their geographical locations, years of experience in medical practice, and inheritance of their medical knowledge (Table 5).

## 4. Conclusions

This study investigated traditional treatments, pairing herbs used with PPvY in therapy and processing methods of PPvY through interviews with folk doctors in northwest Yunnan, China. The survey recorded the clinical applications of PPvY among fourteen folk doctors in northwest Yunnan, which revealed twenty-three traditional treatments, thirty treatments pairing herbs with PPvY in therapy, and eight processing methods of PPvY. These results reflected the diversity of medicinal plants and the wealth of traditional medical knowledge in the northwest region of Yunnan. The most common traditional efficacies of PPvY included anti-cancer, anti-inflammatory, and clearing heat and detoxifying. The TCM herbs most frequency used in conjunction with PPvY were *E. sinensis* and *G. yunnanensis*. The commonly used processing methods for PPvY primarily involved both dry and fresh forms, while special processing methods, such as processing in wine and honey, steaming, and foil-packet boiling, were worth further research. However, with the rapid development of the economy, traditional medical knowledge and medicinal plants have been threatened for various reasons in recent years. Therefore, policies and practices aimed at protecting these medicinal plants and the associated traditional medical knowledge are necessary. In the future, five key aspects should be prioritized for the conservation and sustainable use of medicinal plants: (1) Preservation of traditional knowledge: the first step is to document and protect local traditional medicinal plant knowledge while promoting its rational utilization. (2) Scientific research and technological development: Based on the growth properties and commonly used parts of medicinal plants, it is essential to develop new medicinal parts and innovative uses. Additionally, reasonable and sustainable collection times and methods should be established. For example, PPvY, a perennial herbaceous plant, has anti-cancer activity in its aboveground parts [61]. Thus, these parts can serve as a substitute for rhizomes, helping to protect PPvY resources and promote sustainable utilization. (3) Cultivation techniques: to develop artificial propagation and cultivation techniques for medicinal plants to reduce reliance on and replace the collection of wild plants. (4) Policy formulation: It is crucial to formulate relevant policies that promote an easily accessible and benefit-sharing system for local traditional medical knowledge. (5) Monitoring system: finally, establishing a monitoring system for medicinal plant resources will allow for regular evaluation of their status and ecological impact.

## Figures and Tables

**Table 1:** Diseases treated with formulas containing PPvY.

Diseases		D1	D2	D3	D4	D5	D6	D7	D8	D9	D10	D11	D12	D13	D14
Cancer	Lung cancer								Y	Y	Y	Y			
	Liver cancer								Y	Y	Y				
	Gastric cancer											Y			
	All cancer								Y		Y				Y
Anti-inflammatory			Y	Y	Y	Y	Y		Y	Y	Y	Y	Y	Y	Y
Clearing heat and detoxifying		Y				Y			Y	Y	Y				
Respiratory system diseases	Pulmonary diseases	Y		Y											
	Tonsillitis		Y									Y			
	Sore throat					Y									
	Tracheitis							Y							
	Pharyngitis							Y							
	Cold	Y													
Skin and subcutaneous tissue diseases	Traumatic injury			Y				Y	Y	Y	Y				
	Furuncles, carbuncles, abscesses						Y		Y	Y	Y				
	Hemorrhoids							Y							
	Ulceration						Y								
	Snake venom						Y								
Other types of diseases	Mumps			Y	Y	Y	Y					Y			
	Infantile syncope due to fright	Y													
	Gastrointestinal disease	Y													
	Thyroiditis			Y											
	Gynecological diseases			Y											
	Clearing damp									Y					

**Table 2:** Other herbs used in formulas with PPvY.

TCM Name in Pinyin	Scientific Name	D1	D2	D3	D4	D5	D6	D7	D8	D9	D10	D11	D12	D13	D14
Ma Huang	*Ephedra likiangensis* Florin	Y				Y									
Yun Nan Gan Cao	*Glycyrrhiza yunnanensis* S. H. Cheng and L. K. Dai ex P. C. Li	Y	Y	Y	Y		Y			Y					
Zhu Jun	*Engleromyces sinensis* M.A. Whalley, Khalil, T.Z. Wei, Y.J. Yao and Whalley			Y	Y	Y	Y		Y	Y	Y	Y	Y	Y	Y
Du Suan Lan	*Pleione bulbocodioides* (Franch.) Rolfe			Y			Y		Y	Y					
Tian Ma	*Gastrodia elata* Bl.					Y	Y		Y	Y					
Zhu Zi Shen	*Panax bipinnatifidus* Seem.					Y		Y			Y				Y
Hua Shu Rong	*Polyporus obliquus* Fr.						Y								
Jin Tie Suo	*Psammosilene tunicoides* W. C. Wu and C. Y. Wu							Y		Y					
Cao Wu	*Aconitum kusnezoffii* Rehder							Y							
Xue Shang Yi Zhi Hao	*Aconitum brachypodum* var. laxiflorum H. R. Fletcher and Lauener							Y							
San Qi	*Panax notoginseng* (Burkill) F. H. Chen ex C. H. Chow							Y							
Sha Qi	*Rheum delavayi* Franch.										Y				
Tian Nan Xing	*Arisaema erubescens* (Wall.) Schott										Y				
Yan Bai Zhi	*Heracleum likiangense* Wolff										Y				
Tu Dang Gui	*Angelica decursiva* (Miq.) Franch. and Sav.										Y				
Qin Jiao	*Gentiana macrophylla* Pall.										Y				
Pu Gong Ying	*Taraxacum mongolicum* Hand.-Mazz.										Y				Y
Du Huo	*Angelica pubescens* Maxim. f. *biserrata* Shan et Yuan										Y				
Qian Hu	*Peucedanum praeruptorum* Dunn.										Y				
Hu Zhang Cao	*Anemone rivularis* Buch.-Ham. ex DC.								Y	Y					
Bai Hua She She Cao	*Scleromitrion diffusum* (Willd.) R. J. Wang								Y						
Mu Li	*Ostrea gigas* Thunberg											Y			
Chuan Bei Mu	*Fritillaria cirrhosa* D. Don														Y
Bai Zhi	*Angelica dahurica* (Fisch. ex Hoffm.) Benth. and Hook. f. ex Franch. and Sav.														Y
Mao Zhao Cao	*Ranunculus ternatus* Thunb.														Y
Xia Ku Cao	*Prunella vulgaris* L.														Y
Zhu Huang Jun	*Shiraia bambusicola* Henn.									Y					
Dang Gui	*Angelica sinensis* (Oliv.) Diels									Y					
Fo Shou	*Citrus medica* var. *sarcodactylis* (Hoola van Nooten) Swingle									Y					
Qing Yang Shen	*Cynanchum otophyllum* C. K. Schneid.									Y					

**Table 3:** Processing methods for PPvY.

Processing Methods	D1	D2	D3	D4	D5	D6	D7	D8	D9	D10	D11	D12	D13	D14
Use in the fresh	Y	Y	Y	Y	Y	Y	Y	Y	Y	Y				
Dry in the shade			Y		Y			Y	Y	Y				
Dry in the sun						Y	Y			Y				
Processed with honey			Y					Y		Y				
Processed with wine			Y			Y	Y	Y	Y	Y				
Steaming		Y		Y										
Use in the dry	Y	Y		Y							Y	Y	Y	Y
Foil packet boiled										Y				

**Table 4:** ICF values of TCM used by folk doctors.

Diseases or Traditional Efficacy	Total Number of TCM Species (*Nur*)	The Simultaneous Utilized Number of TCM Species (*Nt*)	ICF
Lung cancer	4	1	1
Uterine cancer	9	2	0.88
All cancer	5	2	0.75
Inflammation	3	2	0.5
Pulmonary diseases	7	2	0.83
Various nodules	8	1	1
Infantile syncope due to fright	2	2	0
Mumps	2	2	0
Furuncles, carbuncles, abscesses	5	1	1
Traumatic injury	4	1	1

**Table 5:** Background information of interviewed TCM practitioners.

Folk Doctor	Snow Mountain	Years of Experience	Generations of Doctors	Ethnic Group	Location
1	Haba Snow Mountain	52	3	Naxi	Baidi Village, Sanba Township, Shangri La City, Diqing Prefecture, Yunnan Province
2	Yulong Snow Mountain	53	3	Naxi	Lunan Village, Longpan Township, Yulong County, Lijiang City, Yunnan Province
3	Laojun mountain	28	3	Naxi	Ale Village, Judian Town, Yulong County, Lijiang City, Yunnan Province
4	Laojun mountain	54	3	Naxi	Jinhe Village, Judian Town, Yulong County, Lijiang City, Yunnan Province
5	Laojun mountain	33	3	Naxi	Xinzhu Village, Ludian Township, Yulong County, Lijiang City, Yunnan Province
6	Laojun mountain	42	4	Naxi	Ludian Village, Ludian Township, Yulong County, Lijiang City, Yunnan Province
7	Laojun mountain	50	3	Naxi	Ludian Village, Ludian Township, Yulong County, Lijiang City, Yunnan Province
8	Laojun mountain	52	3	Bai	Lanxiang Village, Shitou Township, Yulong County, Lijiang City, Yunnan Province
9	Laojun mountain	28	4	Bai	Lanxiang Village, Shitou Township, Yulong County, Lijiang City, Yunnan Province
10	Laojun mountain	17	4	Naxi	Jinhe Village, Judian Town, Yulong County, Lijiang City, Yunnan Province
11	Yulong Snow Mountain	65	19	Naxi	Wuyi Street, Wenzhi Lane, Gucheng District, Lijiang City, Yunnan Province
12	Laojun mountain	45	3	Naxi	Judian Town, Yulong County, Lijiang City, Yunnan Province
13	Laojun mountain	27	3	Naxi	Ludian Town, Yulong County, Lijiang City, Yunnan Province
14	Yulong Snow Mountain	19	2	Yi	Xiangshan Comprehensive Market, Yulong County, Lijiang City, Yunnan Province

## Data Availability

The original contributions presented in the study are included in the article, further inquiries can be directed to the corresponding author.

## Abbreviations

PPvY, *Paris polyphylla* var. *yunnanensis* (Franch.) Hand. –Mazz.; TCM, Traditional Chinese Medicine; GAP, good agriculture practice; COVID-19, Corona Virus Disease 2019; SARS, Severe Acute Respiratory Syndrome; Dn, the number for folk doctors (n = 1 to 14); ICF, Informant consensus factor.
